# Supplementary material for: Emergence of Calabi–Yau manifolds in high-precision black-hole scattering
Source: Nature. 2025 May 14;641(8063):603–7. doi: 10.1038/s41586-025-08984-2 (PMC12078182; doi:10.1038/s41586-025-08984-2)
Supplement: Supplementary file 1 — Supplementary Information [file 41586_2025_8984_MOESM1_ESM.pdf]

nature portfolio

https://doi.org/10.1038/s41586-025-08984-2

Supplementary information

Emergence of Calabi–Yau manifolds in high-precision black-hole scattering

In the format provided by the authors and unedited

SUPPLEMENTARY INFORMATION

I. FURTHER DETAILS OF RESULTS

The main result of our work is the 5PM-1SF component of the momentum impulse Δp_1^μ, from which the corresponding components of the scattering angle θ, loss of energy $E_{\rm rad}$ and recoil $\mathbf{P}_{\rm recoil}$ may subsequently be derived. The 5PM-1SF impulse is parametrized by eight coefficients: $c_{b,{\rm even/odd}}(\gamma)$, $c_{v,{\rm even/odd}}(\gamma)$, $\bar{c}_{b,{\rm even/odd}}(\gamma)$ and $\bar{c}_{v,{\rm even/odd}}(\gamma)$, plus the simpler $c'_v(\gamma)$ and $\bar{c}'_v(\gamma)$ coefficients that are pre-determined by lower-PM results. In Eq. (23), the former coefficients were expanded in terms of γ-dependent functions $F_{b/v,{\rm even/odd}}(\gamma)$ multiplied by polynomials $d_{b/v,{\rm even/odd}}(\gamma)$. The functions $F_{b,{\rm even/odd}}$ are simpler than $F_{v,{\rm even/odd}}$ due to the absence of K3 and CY3 periods in the former, the only integration kernels within the iterated integrals (4) being x^{-1}, $(1\pm x)^{-1}$ and $x/(1+x^2)=((x+i)^{-1}+(x-i)^{-1})/2$ — i.e. simple poles. These functions may therefore be expressed in terms of multiple polylogarithms (MPLs):

$$G(a_1,\ldots,a_n;y)=\int_0^y\frac{{\rm d}y'}{y'-a_1}G(a_2,\ldots,a_n;y')\,. \tag{S.1}$$

In order to apply a conventional definition of MPLs, for convenience here we have introduced $y=1-x$. The letters a_i are then shifted to $\{0,1,2,1\pm {\rm i}\}$, with the two imaginary letters occurring always in pairs. All expansion functions of the b coefficients $F_{b,{\rm even/odd}}(\gamma)$ (and their $\overline{\rm 1SF}$ counterparts) are linear combinations of MPLs of this kind up to weight 3.

Besides these same MPLs (up to weight-3), the functions $F_{v,{\rm even}}(\gamma)$ (and $\overline{\rm 1SF}$) also involve the K3 period $\varpi_{0,{\rm K3}}(\gamma)$ and its derivatives $\varpi'_{0,{\rm K3}}(\gamma)$, $\varpi''_{0,{\rm K3}}(\gamma)$. The K3 period also occurs within the following three iterated integrals (4):

$$\mathcal{I}\left(\frac{1+x^2}{x}\varpi_{0,{\rm K3}}(x);x\right)\,, \tag{S.2a}$$

$$\mathcal{I}\left(\frac{1}{(1-x^2)x\,\varpi_{0,{\rm K3}}(x)},\frac{1+x^2}{x}\varpi_{0,{\rm K3}}(x);x\right)\,, \tag{S.2b}$$

$$\mathcal{I}\left(\frac{1}{(1-x^2)x\,\varpi_{0,{\rm K3}}(x)},\frac{1}{(1-x^2)x\,\varpi_{0,{\rm K3}}(x)},\frac{1+x^2}{x}\varpi_{0,{\rm K3}}(x);x\right)\,. \tag{S.2c}$$

Finally, $F_{v,{\rm odd}}(\gamma)$ (and $\overline{\rm 1SF}$) depend on both the K3 and CY3 periods, $\varpi_{0,{\rm K3}}(\gamma)$ and $\varpi_0(\gamma)$, their derivatives $\varpi_{0,{\rm K3}}(\gamma)$, $\varpi'_{0,{\rm K3}}(\gamma)$, $\varpi'_0(\gamma)$, $\varpi''_0(\gamma)$ and $\varpi'''_0(\gamma)$, plus $\alpha(x)$ and $\alpha'(x)$. There are also now iterated integrals $\mathcal{I}$ whose kernels depend on the CY3 and K3 periods and $\alpha(x)$, with up to four iterations. Finally, we encounter all the $G_i(\gamma)$ functions given below in Eqs. (S.53), which are also iterated integrals on kernels that depend on the periods and $\alpha(x)$ and their derivatives.

In all coefficients except $c_{b,{\rm odd}}$, $\bar{c}_{b,{\rm odd}}$ and $c_{v,{\rm even}}$, a logarithm $\log(\gamma-1)$ is present in the results, and no other functions have a logarithmic divergence in the limit $\gamma\to1$. When using MPLs, we choose basis functions that are free of logarithms in this limit. For the iterated integrals of CY3 and K3 periods, however, we have no general procedure for identifying such logarithms. Wherever possible, we have collected iterated integrals together in linear combinations so that logarithms of $\gamma-1$ cancel in the $\gamma\to1$ limit, but this was not possible in all cases. When one collects all terms, however, such cancellations do occur. Finally, and as a general rule, functions $F_{b/v,{\rm even/odd}}(\gamma)$ are chosen with a definite parity $v\to-v$ wherever possible. This follows the number of radiative gravitons and is equivalent to $x\to1/x$ or $\sqrt{\gamma^2-1}\to-\sqrt{\gamma^2-1}$. For the more advanced basis functions, with iterated integrals involving the CY3 and K3 periods, we were not always able to construct basis functions with the symmetry property — a complete and systematic organization of these functions is left for future work.

A. Scattering angle

The 5PM component of the relative scattering angle depends only on the b-type coefficients of the 5PM impulse, and may therefore be expressed solely in terms of MPLs — as was the case for its conservative even-in-v part [7]. The relevant coefficients of Eq. (6), determined by our 5PM 1SF and $\overline{\rm 1SF}$ results, are $\theta^{(5,1)}(\gamma)$ and $\theta^{(5,3)}(\gamma)$. Their contribution from an even or odd number of radiative gravitons, $\theta^{(5,1)}_{\rm even/odd}(\gamma)$ and $\theta^{(5,3)}_{\rm even/odd}(\gamma)$, are also, respectively, even and odd with respect to the symmetry $v\to-v$. In a PN velocity expansion on has

$$\theta^{(5,1)}=\frac{4}{5v^8}-\frac{137}{5v^6}+\frac{3008}{45v^5}+\frac{\frac{41\pi^2}{4}-\frac{3427}{6}}{v^4}+\frac{\frac{84\pi^2}{5}-\frac{4096}{1575}}{v^3}+\frac{-\frac{12544\log(2v)}{45}+\frac{3593\pi^2}{72}-\frac{445867}{432}}{v^2}+\frac{\frac{2144536}{11025}+\frac{453\pi^2}{35}}{v}$$

$$+\left(-\frac{7552\log(2v)}{1575}+\frac{246527\pi^2}{1440}-\frac{1111790903}{756000}\right)+\left(\frac{19424344}{363825}+\frac{1787\pi^2}{672}\right)v$$
$$+\left(-\frac{1762784\log(2v)}{11025}-\frac{184881\pi^2}{2240}+\frac{56424801733}{49392000}\right)v^2+\left(\frac{16004496043}{104053950}-\frac{835619\pi^2}{59136}\right)v^3+\mathcal{O}(v^4)\,, \tag{S.3a}$$
$$\theta^{(5,3)}=\frac{128}{3v}+\left(\frac{14528}{175}+\frac{37\pi^2}{10}\right)v-\frac{22016v^2}{225}+\left(\frac{3262832}{33075}+\frac{893\pi^2}{112}\right)v^3+\frac{2877184v^4}{7875}+\left(\frac{15803\pi^2}{960}-\frac{4464536}{14553}\right)v^5$$
$$+\frac{970766528v^6}{1819125}+\left(\frac{11234077\pi^2}{394240}-\frac{258810752887}{780404625}\right)v^7+\frac{83694772064v^8}{70945875}$$
$$+\left(\frac{119425757\pi^2}{2795520}-\frac{5667010769993}{5533778250}\right)v^9+\frac{11015320038116v^{10}}{5462832375}+\mathcal{O}(v^{11})\,. \tag{S.3b}$$

As discussed in Methods, the PN expansion of $\theta^{(5,1)}$ has been checked up to and including v^1 against known PN data. The complete expressions are provided in the `observables.m` file of the repository submission. Here the MPLs may be PN expanded using conventional tools, such as `PolyLogTools` [8], which is why we do not provide explicit v-expansions there.

B. Radiated momentum

The 5PM-1SF component of the loss of four-momentum $P^\mu_{\rm rad}$ (24) is given by the impulse coefficients via

$$r_1(\gamma)=c_b(\gamma)-\bar{c}_b(\gamma)\,, \tag{S.4a}$$
$$r_2(\gamma)=\frac{c_v(\gamma)-\bar{c}_v(\gamma)-c'_v(\gamma)+\bar{c}'_v(\gamma)}{2(\gamma-1)}\,, \tag{S.4b}$$
$$r_3(\gamma)=-\frac{c_v(\gamma)+\bar{c}_v(\gamma)+c'_v(\gamma)+\bar{c}'_v(\gamma)}{2(\gamma+1)}\,. \tag{S.4c}$$

Thus, r_1 depends only on MPLs and $r_{2,3}$ have the full generality of $c_{v,\rm odd}(\gamma)$ and $\bar{c}_{v,\rm odd}(\gamma)$. These results are collected in the `observables.m` file of the repository submission. In the centre-of-mass frame, wherein $P^\mu_{\rm rad}=(E_{\rm rad},\mathbf{P}_{\rm recoil})$, the 5PM components take the form ($\Gamma=\sqrt{1+2\nu(\gamma-1)}$, $\nu=\mu/M=m_1m_2/M^2$, $M=m_1+m_2$)

$$E^{(5)}_{\rm rad}=\frac{M^6\nu^2}{\Gamma b^5}\left[(1-\gamma)r_2(\gamma)+(1+\gamma)r_3(\gamma)+\mathcal{O}(\nu)\right]\,, \tag{S.5}$$
$$\mathbf{P}^{(5)}_{\rm recoil}=\frac{M^5\nu^2(m_1-m_2)}{b^5}\Big(r_1(\gamma)\hat{\mathbf{b}}+(r_2(\gamma)-r_3(\gamma))\frac{\mathbf{p}_{\rm in}}{\mu}+\mathcal{O}(\nu)\Big)\,. \tag{S.6}$$

An explicit formula for $E^{(5)}_{\rm rad}$ up to v^9 was provided in Methods (27). The PN-expanded components of the recoil are

$$r_1(\gamma)=-\frac{64}{3v^2}-\frac{16192}{525}-\frac{37\pi^2}{20}-\frac{30208v}{225}+\left(-\frac{856768}{33075}-\frac{3429\pi^2}{1120}\right)v^2-\frac{22016v^3}{2625}+\left(-\frac{1117888}{4851}-\frac{80723\pi^2}{13440}\right)v^4$$
$$-\frac{123897344v^5}{606375}+\left(-\frac{36746586176}{780404625}-\frac{22515319\pi^2}{2365440}\right)v^6-\frac{16343148032v^7}{70945875}+\left(-\frac{169791059264}{869593725}-\frac{33021283\pi^2}{2562560}\right)v^8$$
$$-\frac{584895938048v^9}{1820944125}+\left(-\frac{53344474395584}{517408266375}-\frac{7894087273\pi^2}{492011520}\right)v^{10}+\mathcal{O}(v^{11})\,, \tag{S.7}$$

and

$$\frac{r_2(\gamma)-r_3(\gamma)}{\pi}=-\frac{53}{3v^3}+\frac{72997}{5040v}+\frac{1491}{400}-\frac{1509\pi^2}{140}+\frac{20211v}{640}+\left(\frac{75661\pi^2}{4480}-\frac{2678867}{16800}\right)v^2$$
$$+\left(\frac{41053\log\left(\frac{v}{2}\right)}{2450}-\frac{503\pi^2}{70}-\frac{123069432361}{4346496000}\right)v^3+\left(\frac{6139957\pi^2}{394240}-\frac{15259259693}{124185600}\right)v^4$$
$$+\left(\frac{223443793\log\left(\frac{v}{2}\right)}{15523200}-\frac{69203\pi^2}{6720}+\frac{64307545227137}{22375761408000}\right)v^5+\left(\frac{252585041\pi^2}{5857280}-\frac{16101198460801}{56504448000}\right)v^6$$

$$+\left(\frac{313945836331\log\left(\frac{v}{2}\right)}{8879270400}-\frac{346561\pi^2}{16896}-\frac{204989896406483131}{1828419360768000}\right)v^7+\left(\frac{918930349\pi^2}{11714560}-\frac{2985881877364537}{8524099584000}\right)v^8$$
$$+\left(\frac{16292858440697\log\left(\frac{v}{2}\right)}{307814707200}-\frac{288050119\pi^2}{8785920}-\frac{431015900890630345739}{1676186519175168000}\right)v^9$$
$$+\left(\frac{326238718783\pi^2}{2788065280}-\frac{984733411903585726667}{217581903931392000}\right)v^{10}+\mathcal{O}(v^{11})\,. \tag{S.8}$$

As discussed in Methods, the first six terms of each series were checked against known PN data the PN series of E_{rad} and the $\hat{\mathbf{b}}$ and $\mathbf{p}_{\mathrm{in}}$ components of $\mathbf{P}_{\mathrm{recoil}}$. We provide the PN expansion of the impulse in the velocity direction explicitly up to $\mathcal{O}(v^{500})$ in the repository submission.

II. GRAVITON GAUGE FIXING

In order to gauge fix the bulk graviton action, we begin by rewriting the Einstein-Hilbert term of Eq. (1) via a total derivative and augment it with a gauge-fixing term:

$$\mathcal{S}_{\mathrm{bulk}}=-\frac{1}{16\pi G}\int \mathrm{d}^4x\Big[\sqrt{-g}g^{\alpha\beta}g^{\gamma\delta}g^{\mu\nu}(\Gamma_{\alpha\gamma\delta}\Gamma_{\mu\beta\nu}-\Gamma_{\alpha\gamma\mu}\Gamma_{\delta\beta\nu})+\tfrac{1}{2}G^\mu G^\nu\eta_{\mu\nu}\Big]\,. \tag{S.9}$$

We employ a non-linearly extended de Donder gauge using the gauge fixing vector G^μ, the graviton field $h_{\mu\nu}$ (absorbing the coupling $\sqrt{32\pi G}$), and its trace $h=h_\mu{}^\mu$ of the form

$$\begin{aligned}G^\mu=&\partial_{\nu_1}h^{\mu\nu_1}-\tfrac{1}{2}\partial^\mu h+\tfrac{1}{2}\partial_{\nu_1}h\,h^{\mu\nu_1}+\tfrac{1}{2}\partial^\mu h_{\nu_1\nu_2}\,h^{\nu_1\nu_2}-\partial^{\nu_1}h^{\mu\nu_2}h_{\nu_1\nu_2}-\tfrac{1}{4}\partial^\mu h\,h\\&+\partial_{\nu_1}h^{\nu_3\mu}h^{\nu_1\nu_2}\,h_{\nu_2\nu_3}+\tfrac{1}{2}\partial_{\nu_1}h^{\nu_1\nu_2}\,h_{\nu_2\nu_3}\,h^{\nu_3\mu}-\tfrac{1}{4}\partial_{\nu_2}h\,h_{\nu_2\nu_3}\,h^{\nu_3\mu}+\partial_{\nu_1}h_{\nu_2\nu_3}\,h^{\nu_1\nu_2}h^{\nu_3\mu}\\&-\tfrac{1}{2}\partial^{\nu_2}h_{\nu_1\nu_2}\,h^{\mu\nu_1}\,h+\tfrac{1}{4}\partial^\mu h_{\nu_1\nu_2}\,h^{\nu_1\nu_2}h-\tfrac{1}{2}\partial^{\nu_1}h^{\mu\nu_2}\,h_{\nu_1\nu_2}h+\tfrac{1}{8}\partial_{\nu_1}h^{\mu\nu_1}\,h^2\,.\end{aligned} \tag{S.10}$$

The quadratic-in-$h_{\mu\nu}$ term arising in (S.9) yields the graviton propagator of Fig. 3, whereas the non-linear gauge fixing terms are engineered in such a fashion as to maximally simplify the cubic and quartic graviton vertices. The resulting Feynman vertices up to six-graviton legs along with the used worldline vertices are provided in the `feynman rules.m` file.

III. EXPANSION OF DIFFERENTIAL EQUATION AND SINGLE POLES

In the Methods section, we have constructed a canonical basis satisfying an ϵ-factorized differential equation:

$$\frac{\mathrm{d}}{\mathrm{d}x}\vec{J}(x;\epsilon)=\epsilon\hat{A}(x)\,\vec{J}(x;\epsilon)\,. \tag{S.11}$$

Besides the factorization of ϵ in the differential equation (S.11), an important property of a canonical basis is that (S.11) has only simple poles. This guarantees that we only have logarithmic singularities after integration. In other words, when we expand the matrix $\hat{A}(x)$ around all its singularities, i.e., for $x=0,1,\infty$, we find at most simple poles. To test this property, we have to take into account the series expansions of the new transcendental functions $\varpi_{0,\mathrm{K3}},\varpi_0,\varpi_1,\alpha_1$, and G_i — defined only by the differential equations they satisfy, and thus leaving an ambiguity in their choice. For this discussion, it is sufficient to study the differential equation (S.11) around $x=0$, where we have derived the canonical form, and $x=1$, around which we solve the differential equation to compute the velocity expansion. Around both singularities, we provide our local choices of new transcendental functions — not the proper analytic continuations of these objects. In this way, we guarantee that we only exhibit single poles and that all entries of $\hat{A}(x)$ have a sensible series expansion so that corresponding iterated integrals are easily computed.

The series expansions of our functions are as follows. Around $x=0$, the CY3 has a special property known as the point of maximal unipotent monodromy. At this point, the basis of periods shows a hierarchy in the number of appearing logarithms, i.e., $\varpi_i^{[0]}\sim\log^i(x)$ for $i=0,1,2,3$. From this structure, it is quite natural to choose

$\varpi_0^{[0]}, \varpi_1^{[0]}, \alpha_1^{[0]}$ in the following way:

$$\begin{aligned}
\varpi_0^{[0]} &= x + \frac{x^5}{16} + \frac{81x^9}{4096} + \dots\,, \\
\varpi_1^{[0]} &= \log(x)\left(x + \frac{x^5}{16} + \frac{81x^9}{4096} + \dots\right) + \frac{x^5}{16} + \frac{189x^9}{8192} + \dots\,, \\
\alpha_1^{[0]} &= 1 - \frac{x^4}{4} - \frac{93x^8}{1024} + \dots\,.
\end{aligned} \tag{S.12}$$

Around $x = 1$, the solution space of periods has a different structure containing three power series solutions and just a single logarithmic one. We pick $\varpi_0^{[1]}, \varpi_1^{[1]}$ as a linear combination of these three power series solutions:

$$\begin{aligned}
\varpi_0^{[1]} &= 1 - (1-x) + \frac{(1-x)^3}{3} + \frac{(1-x)^4}{3} + \dots\,, \\
\varpi_1^{[1]} &= 1 - \frac{(1-x)^2}{2} - \frac{(1-x)^3}{6} + \frac{(1-x)^4}{3} + \dots\,, \\
\alpha_1^{[1]} &= 1 - 2\cdot(1-x) + 2\cdot(1-x)^2 - \frac{(1-x)^3}{3} + \frac{(1-x)^4}{2} + \dots\,.
\end{aligned} \tag{S.13}$$

Another (equally valid) choice would change the boundary values of our integrals but not the final physical results. The K3 surface, however, is special in that it has at both singularities $x = 0$ and $x = 1$: a point of maximal unipotent monodromy. Therefore, at both points, there is a natural choice for the period $\varpi_{0,\mathrm{K3}}$. We simply take the unique power series solution, which we can also express through squares of elliptic integrals:

$$\begin{aligned}
\varpi_{0,\mathrm{K3}}^{[0]} &= \frac{4}{\pi^2}K^2(x^2) &&= 1 + \frac{x^2}{2} + \frac{11x^4}{32} + \dots\,, \\
\varpi_{0,\mathrm{K3}}^{[1]} &= \frac{4}{\pi^2}K^2(1-x^2) &&= 1 - (1-x) + \frac{7(1-x)^2}{8} - \frac{3(1-x)^3}{4} + \dots\,.
\end{aligned} \tag{S.14}$$

Given these prerequisites, we may now series expand the differential equation in $(1-x)$:

$$\hat{A} = \sum_{j=0}^{2} \hat{A}_{-1,j} \frac{\log^j(1-x)}{1-x} + \sum_{i,j\geq 0} \hat{A}_{i,j}(1-x)^i \log^j(1-x)\,. \tag{S.15}$$

We see immediately that there are no higher poles appearing. The terms $(1-x)^{-1}\log^j(1-x)$ for $j = 1, 2$ are also valid because, after integration, they give rise only to logarithmic singularities. These terms arise from the new G_i functions and are consequently associated with the CY3. This series-expanded form of the differential equation (S.11) plays an important role in our matching of boundary integrals to the slow-velocity $x \to 1$ limit.

IV. BOUNDARY MATCHING

The solution to Eq. (S.11) is a path-ordered exponential:

$$\vec{J}(x;\epsilon) = \mathcal{P}e^{\epsilon\int_1^x \mathrm{d}x \hat{A}(x)}\vec{j}\,, \tag{S.16}$$

where the vector $\vec{j}$ contains the boundary values of our integrals at $x = 1$, coinciding with the small-velocity limit. This form can be expanded in ϵ up to the required order and naturally gives rise to the iterated integrals defined in Eq. (4) of the main text. Our task is now to fix the boundary constants $\vec{j}$ in the small-velocity $x \to 1$ limit, wherein our master integrals become trivial in x but are still non-trivial in ϵ — see Section V. While in principle we could compare every integral with its $x \to 1$ limit, we seek to minimize the number of boundary integrals that must be performed explicitly. Accordingly, we employ a strategy of imposing analyticity constraints on the general solution of Eq. (S.11) — relating various boundary values of the master integrals with each other, and leaving only a small number of unfixed boundary constants. This is done by also solving the differential equation (S.11) perturbatively in $(1-x)$, while retaining its all-order dependence on ϵ:

$$\vec{J}(x;\epsilon) = \sum_{k,m,n} \vec{f}_{k,m,n}(\epsilon)(1-x)^{k+m\epsilon}\log^n(1-x)\,. \tag{S.17}$$

The boundary constants are now contained within the functions $\vec{f}_{k,m,n}(\epsilon)$, and we can easily go from (S.17) to (S.16) by expanding in ϵ.

The ϵ-resummed solution (S.17) is derived from the velocity-expanded form of the differential equation (S.15), following Wasow's method [2]. First, we compute the leading order in $(1-x)$, i.e. we set $k=0$ and keep only the terms $\hat{A}_{-1,i}$ for $i=0,1,2$ in Eq. (S.15). In this step, we also determine the range of m in (S.17). We write $\vec{J} = \hat{S}_0(x;\epsilon)\cdot\vec{j} + \mathcal{O}((1-x))$ such that $\hat{S}_0(x;\epsilon)$ contains all the $(1-x)^{m\epsilon}$ scalings. Then, $\hat{S}_0$ is determined by:

$$\frac{\mathrm{d}\hat{S}_0(x;\epsilon)}{\mathrm{d}x} = \epsilon\sum_{j=0}^{2}\hat{A}_{-1,j}\,\frac{\log^j(1-x)}{1-x}\,\hat{S}_0(x;\epsilon)\,. \tag{S.18}$$

Only the entries of $\hat{A}(x)$ that describe couplings to the CY3 sector contain powers of $\log(1-x)$ in their expansions. To solve the differential equation (S.18) and compute $\hat{S}_0$, we therefore split our integrals into three groups: (i) those that do not couple to the CY3 sector (in the top-left corner of Fig. 5, above the Calabi-Yau integrals); (ii) integrals in the CY3 sector; (iii) all other integrals coupling through the differential equation to the CY3 sector. For the first group, $\hat{A}_{-1,1}=\hat{A}_{-1,2}=0$ and so Eq. (S.18) is solved by the matrix exponential $(1-x)^{\epsilon\hat{A}_{-1,0}}$.[1] For the CY3 sector, we solve (S.18) explicitly using `Mathematica`'s `DSolve` command. For the third group, their homogeneous part is once again given by a matrix exponential since $\hat{A}_{-1,1},\hat{A}_{-1,2}$ are all vanishing. For the inhomogeneous part, we use the method of variation of constants. In this way, we have determined $\hat{S}_0$: the possible $(1-x)$ scalings from Eq. (S.17) are $m\in\{10,8,2,0,-2,-4,-6\}$.

We now continue and compute more terms in the $(1-x)$ expansion of Eq. (S.17). The summation range of m is completely fixed by the leading order in $(1-x)$, whereas new powers of $\log(1-x)$ can appear at higher orders in the $(1-x)$ expansion. Therefore, we rewrite the solution (S.17), splitting the previously derived leading-order contributions from higher-order terms:

$$\vec{J} = \sum_{i,j}^{N}\left(\hat{F}_{i,j}(\epsilon)(1-x)^i\log^j(1-x)\right)\cdot\hat{S}_0(x;\epsilon)\cdot\vec{j} + \mathcal{O}\left((1-x)^{N+1}\right)\,. \tag{S.19}$$

Here, N is the order in which we want to expand our integrals, and we have recollected the constants $\vec{f}_{k,m,n}(\epsilon)$ into the matrix $\hat{F}_{i,j}(\epsilon)$ multiplying $\hat{S}_0(x;\epsilon)$. In this way, we calculate the ϵ-resummed form of our solution to arbitrary order in $(1-x)$.

Having now determined the slow-velocity ϵ-resummed solution, we can begin imposing analyticity constraints to fix boundary values. A general feature of Feynman integrals is that all terms of the form $(1-x)^{m\epsilon}$ with $m>0$ drop out. Intuitively, this is because the only possible origin of such terms is the integration measure $\mathrm{d}^{4-2\epsilon}\ell$ of our integrals — see Fig. 4 of the main text. Since the measure contains only negative powers of ϵ, our integrals cannot have positive ϵ eigenvalues appearing in $\hat{S}_0(x;\epsilon)$ — a more rigorous justification is given in refs. [4, 5]. Next, the method of regions yields more precise statements about the $(1-x)^{m\epsilon}$ terms appearing in the expansions of the initial integrals. Transforming back to the original (non-canonicalized) basis:

$$\lim_{x\to1}\vec{I}(x;\epsilon) = \lim_{x\to1}\hat{T}(x;\epsilon)^{-1}\vec{J}(x;\epsilon)\,. \tag{S.20}$$

Since the rotation $\hat{T}(x;\epsilon)^{-1}$ depends non-trivially on $(1-x)$, we expand Eq. (S.19) to higher orders in $(1-x)$. Each region comes with a different overall velocity scaling $(1-x)^{-2m\epsilon}$, where m is the number of radiative gravitons — scaling as $\ell_i^{\mathrm{R}} = (\ell_i^0,\ell_i)\sim(v,v)$. By requiring that the wrong scalings on each side of Eq. (S.20) cancel, we obtain further relations between the boundary constants. For example, in the (PPP) region, all constants coupling to terms with velocity scaling $m\neq0$ are set to zero. This fixes the majority of our boundary constants.

All remaining boundary constants must now be fixed by comparison with explicit boundary integrals. On these we perform IBP reduction, further reducing the number of manual computations that must be performed. The remaining boundary integrals that we needed to compute are detailed in Section V.

V. BOUNDARY INTEGRALS

Here we describe our strategy for calculating the boundary integrals required to fix the solutions to the differential equations. We applied a number of different methods depending on the boundary integral in question.

[1] To compute the matrix exponential quickly, we use the `Mathematica` package `Libra` [3], which makes use of the block-triangular structure of our differential equation.

A. Nomenclature

Due to the static $v \to 0$ limit, almost all boundary integrals are ladder-type integrals. We now introduce a nomenclature to specify every boundary integral in the planar 1SF sector. Here, three facts about each loop in the planar graphs may be specified:

1. The type of propagator above the loop. This is typically a worldline ω or a graviton bubble g, but it can also be more sophisticated, such as a "tail-of-tail" bubble.
2. The direction of the propagator, which we specify as a subscript to a propagator. This could be Feynman (nothing), retarded $(+)$, advanced $(-)$, or a $-i$ times a cut propagator $(\times)$.
3. The number of "jumps", as a subscript to M. For brevity, this is omitted when there are no jumps.

FIG. 3. The M, M_1, and M_2 families, with zero, one, and two jumps, respectively.

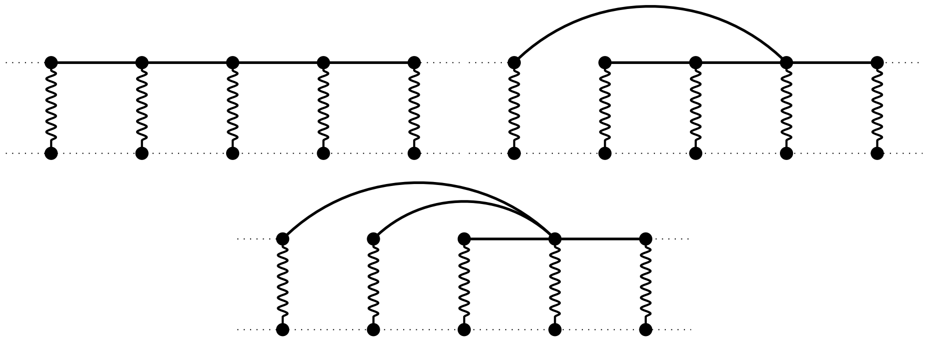

In this notation, the cut identity

$$-i\tilde{\delta}(x) = \frac{1}{x + \mathrm{i}0^+} - \frac{1}{x - \mathrm{i}0^+} \tag{S.21}$$

can be re-expressed as

$$M[\ldots, \omega_\times, \ldots] = M[\ldots, \omega_+, \ldots] - M[\ldots, \omega_-, \ldots] \,. \tag{S.22}$$

In the general case, we must calculate all possible $\mathrm{i}0^+$ routings. Therefore, given an integral calculated with one $\mathrm{i}0^+$ routing and the cut, which factorizes into lower-loop integrals, we obtain the opposite $\mathrm{i}0^+$ routing "for free".

To clarify the nomenclature used in the repository, we give some specific examples. Our convention for the diagrams is that arrows pointing right indicate a retarded $1/(\ell \cdot v + \mathrm{i}0^+)$ propagator, while arrows pointing left also indicate a retarded propagator, but with opposite momentum flow $1/(-\ell \cdot v + \mathrm{i}0^+)$.

$M[g_+ g_+ g_-] =$

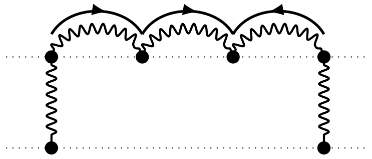

$$= -\frac{1}{12288\pi^4} + \frac{12\gamma_E - 41 - 6\log(2) - 12\log(\pi)}{36864\pi^4}\epsilon + \mathcal{O}(\epsilon^2) \,, \tag{S.23}$$

$M[0, g_+, 0] =$

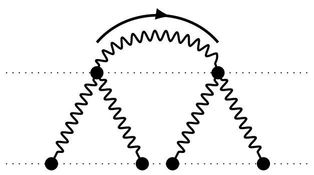

$$= -\frac{1}{65536\pi^2} - \frac{-4\gamma_E + 3 + 26\log(2) + 4\log(\pi)}{65536\pi^2}\epsilon + \mathcal{O}(\epsilon^2) \,, \tag{S.24}$$

$M[\omega_+, g_+, \omega_-] = -$

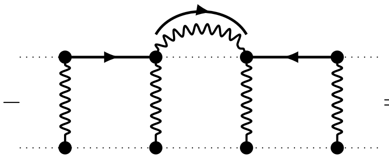

$$= \frac{1}{8192\pi^4\epsilon^2} - \frac{2\gamma_E - 3 - 3\log(2) - 2\log(\pi)}{4096\pi^4\epsilon} + \mathcal{O}(\epsilon^0) \,. \tag{S.25}$$

Note the relative sign in the third line due to the conversion of ω_- to a left-pointing retarded propagator.

B. General Formulae

In the loop-by-loop approach, we principally make use of the following integral, where $\int_\ell = \int \frac{\mathrm{d}^D\ell}{(2\pi)^D}$:

$$\int_\ell \frac{\delta\!\!\!{}^-(\ell\cdot v_1)}{(\ell^2+\mathrm{i}0)^{\nu_1}((\ell-q)^2+\mathrm{i}0)^{\nu_2}(\ell\cdot v_2+\mathrm{i}0)^{\nu_3}} = (4\pi)^{\frac{1-D}{2}}(-1)^{\nu_1+\nu_2+\nu_3} i^{\nu_3}|q|^{D-1-2\nu_1-2\nu_2-\nu_3} \times \frac{\Gamma(\nu_1+\nu_2+\frac{\nu_3}{2}-\frac{D-1}{2})\Gamma(\frac{\nu_3}{2})\Gamma(\frac{D-1}{2}-\nu_1-\frac{\nu_3}{2})\Gamma(\frac{D-1}{2}-\nu_2-\frac{\nu_3}{2})}{2\Gamma(\nu_1)\Gamma(\nu_2)\Gamma(\nu_3)\Gamma(D-1-\nu_1-\nu_2-\nu_3)}, \tag{S.26}$$

where $q\cdot v_1 = q\cdot v_2 = 0$. In practice, this means that a loop-by-loop integration over linear propagators is only possible when a linear propagator is next to a cut. When $\nu_3 = 0$, no such restriction exists, and we use

$$\int_\ell \frac{\delta\!\!\!{}^-(\ell\cdot v_1)}{(\ell^2+\mathrm{i}0)^{\nu_1}((\ell-q)^2+\mathrm{i}0)^{\nu_2}} = (4\pi)^{\frac{1-D}{2}}(-1)^{\nu_1+\nu_2}|q|^{D-1-2\nu_1-2\nu_2}\frac{\Gamma\left(\nu_1+\nu_2-\frac{D-1}{2}\right)\Gamma\left(\frac{D-1}{2}-\nu_1\right)\Gamma\left(\frac{D-1}{2}-\nu_2\right)}{\Gamma(\nu_1)\Gamma(\nu_2)\Gamma(D-1-\nu_1-\nu_2)}. \tag{S.27}$$

For example, $M[\omega_+]$ is a special case of Eq. (S.26) where $\nu_1=\nu_2=\nu_3=1$ (each delta function comes with a factor 2π and each loop integral comes with a factor $1/(2\pi)^4$):

$$M[\omega_+] =$$

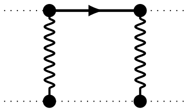

$$= -i(4\pi)^{\epsilon-1}|q|^{-2(\epsilon+1)}\frac{\Gamma(-\epsilon)^2\Gamma(\epsilon+1)}{2\Gamma(-2\epsilon)}. \tag{S.28}$$

Furthermore, in the radiative regions, we encounter "graviton bubbles", which can be integrated out:

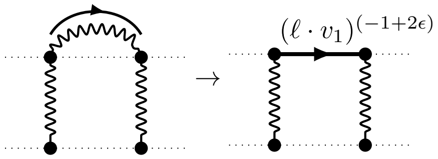

(S.29)

This leads to a linear propagator with an ϵ-dependent, non-integer power:

$$\int_\ell \frac{\delta\!\!\!{}^-(\ell\cdot v)}{(\ell-k)^2+\mathrm{i}0^+\,(k-\ell)\cdot v} = -(+i)^{1-2\epsilon}(k\cdot v+\mathrm{i}0)^{1-2\epsilon}(4\pi)^{\frac{2\epsilon-3}{2}}\Gamma\left(\frac{2\epsilon-1}{2}\right), \tag{S.30}$$

C. Schwinger parametrization

Integrals that cannot be determined using loop-by-loop integration, or fully constrained via cuts and partial fractions, we obtain using Schwinger parametrization. We employ the following identities:

$$\begin{aligned}
\frac{1}{(\omega+\mathrm{i}0^+)^\alpha} &= e^{-i\frac{\pi}{2}\alpha}\frac{1}{\Gamma(\alpha)}\int_0^\infty \mathrm{d}u\, u^{\alpha-1}e^{-u(0-i\omega)}, \\
\frac{1}{(\omega-\mathrm{i}0^+)^\alpha} &= e^{i\frac{\pi}{2}\alpha}\frac{1}{\Gamma(\alpha)}\int_0^\infty \mathrm{d}u\, u^{\alpha-1}e^{-u(0+i\omega)}, \\
\frac{1}{(\boldsymbol{\ell}^2)^\alpha} &= \frac{1}{\Gamma(\alpha)}\int_0^\infty \mathrm{d}u\, u^{\alpha-1}e^{-u\boldsymbol{\ell}^2}.
\end{aligned} \tag{S.31}$$

Prior to Schwinger parametrization, we integrate out all loops possible using Eqs. (S.27) and (S.30). Once the above identities have been applied, integration over loop momenta is trivialized as integrals over Gaussians, leaving behind a non-trivial integral of the Schwinger parameters. There is no systematic procedure for solving these integrals, although we note that `Mathematica` is more effective when integrating over Schwinger parameters corresponding to linear propagators first.

Where graviton bubbles with opposite $\mathrm{i}0^+$ prescription appear on the same loop, e.g. $M[g_+\times g_-]$, we apply the following formula (valid for at least one non-integer power), separating the different prescriptions into separate integrals:

$$\frac{1}{(\omega+\mathrm{i}0^+)^\alpha(\omega-\mathrm{i}0^+)^\beta} = e^{-i\pi\alpha}\frac{\sin[\pi\beta]}{\sin[\pi(\alpha+\beta)]}\frac{1}{(\omega-\mathrm{i}0^+)^{\alpha+\beta}} + e^{i\pi\beta}\frac{\sin[\pi\alpha]}{\sin[\pi(\alpha+\beta)]}\frac{1}{(\omega+\mathrm{i}0^+)^{\alpha+\beta}}. \tag{S.32}$$

A direct corollary of this is that we may convert an arbitrary combination of $\mathrm{i}0^+$ prescriptions in a successive row of graviton jumps and worldline propagators into the sum of two uniform configurations using the formula:

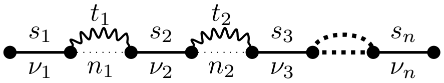

$$= \frac{\sin(\pi\alpha_+)}{\sin(\pi[\alpha_+ + \alpha_-])}$$

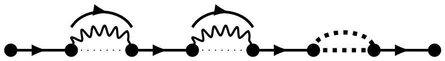

$$+ \frac{\sin(\pi\alpha_-)}{\sin(\pi[\alpha_+ + \alpha_-])}$$

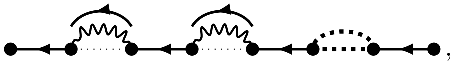

$$, \tag{S.33}$$

where the $s_i = \pm\mathrm{i}0^+$ and $t_i = \pm\mathrm{i}0^+$ yield the $\mathrm{i}0^+$ prescriptions on the left-hand-side and ($D = 4 - 2\epsilon$)

$$\alpha_\pm = \sum_{i|s_i=\pm\mathrm{i}0^+} \nu_i + \sum_{a|t_a=\pm\mathrm{i}0^+} (2n_a - 3 + 2\epsilon)\,. \tag{S.34}$$

Here, we generalized to powers ν_i for the worldline and n_a for the graviton propagators, while in our applications, $\nu_i = n_a = 1$ always.

D. Tail-of-tail integrals

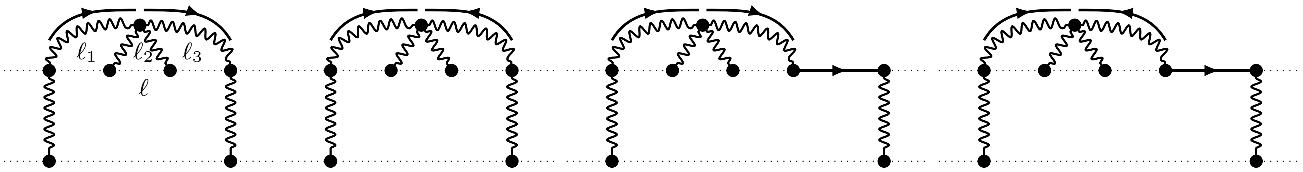

FIG. 4. The required tail-of-tail integrals $M[\mathrm{tail}_+\mathrm{tail}_+]$, $M[\mathrm{tail}_+\mathrm{tail}_-]$, $M[\mathrm{tail}_+\mathrm{tail}_+\omega_+]$, and $M[\mathrm{tail}_+\mathrm{tail}_-\omega_+]$.

A new class of boundary integrals is given by the integrals shown in Fig. 4. We refer to these as tail-of-tail (boundary) integrals following ref. [6]. In total, we require four of those as shown in Fig. 4. We compute these as iterated integrals. Exactly as with the simple graviton bubbles, Eq. S.29, the more complicated "tail-of-tail bubble" depends only on a single scale, its energy. We may therefore integrate this bubble away first resulting in a worldline propagators with an ϵ-dependent power:

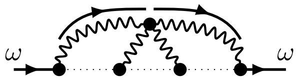

$$= f(\epsilon)(-\mathrm{i}(\omega + \mathrm{i}0^+))^{1-6\epsilon}\,, \tag{S.35}$$

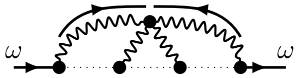

$$= f(\epsilon)(\omega + \mathrm{i}0^+)^{\frac{1-6\epsilon}{2}}(\omega - \mathrm{i}0^+)^{\frac{1-6\epsilon}{2}}\,. \tag{S.36}$$

The $\mathrm{i}0^+$ prescription of the first line is natural: All causality flows naturally from left to right and results in a retarded worldline propagator. Instead, in the second line, the causality flow does not follow the external lines. Due to its symmetry, however, it must be symmetric in retarded and advanced propagators. The powers of propagators on the right-hand-sides are then determined by power counting. On top, a careful analysis shows that the (real) ϵ-dependent factor of proportionality $f(\epsilon)$ is the same when an appropriate factor of i is inserted in the first line.

At this point, having determined $f(\epsilon)$, the remaining integration for the four tail-of-tail boundaries is straightforward. Namely, it is now a one-loop integral with generalized worldline propagators: $M[\omega_+^\alpha \omega_-^\beta]$. The function $f(\epsilon)$ is determined from the three-loop integral,

$$f(\epsilon) = \int_{\boldsymbol{\ell}_1,\boldsymbol{\ell}_2,\boldsymbol{\ell}_3} \frac{1}{(\boldsymbol{\ell}_1 - \boldsymbol{\ell}_2)^2(\boldsymbol{\ell}_2 - \boldsymbol{\ell}_3)^2(\boldsymbol{\ell}_1^2 + 1)(\boldsymbol{\ell}_3^2 + 1)}\,, \tag{S.37}$$

with $(3 - 2\epsilon)$-dimensional Euclidean bold-face momenta.

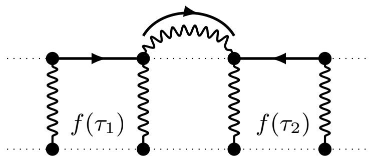

E. Brandenburg Gate integrals

Consider the integral $M[\omega_+, g_+, \omega_-]$, nicknamed "Brandenburg Gate" for its resemblance to the real-world structure. The only loop-by-loop integration we may perform here is the graviton bubble, leaving behind a genuine three-loop integral, for which the integral over Schwinger parameters is too difficult. However, since we only need the first two orders in ϵ, it suffices to find identities at the level of the series expansion. Here, we show how such identities may be derived. In position-space, we can express it as an integral over the time-domain:

$$A = M[\omega_+, g_+, \omega_-]\,(|b|, \epsilon) = \int \mathrm{d}\tau_1 \mathrm{d}\tau_2\, f(\tau_1) G(\tau_1 - \tau_2) f(\tau_2)\,, \tag{S.38}$$

where $f(\tau)$ represents the loops to the side while $G(\tau_1 - \tau_2)$ represents the middle integral including the bubble. Because of $\tau_1 \leftrightarrow \tau_2$ symmetry of the integral, one may assume $G(\tau) = G(-\tau)$. Now consider the following two integrals. By partial fraction identities:

$$\begin{aligned} M[\omega_+, \omega_+, g_+] &= \frac{1}{2} M_1[\omega_+, \omega_+, g_+]\,, \\ M[\omega_-, \omega_-, g_+] &= \frac{1}{2} M_1[\omega_-, \omega_-, g_+]\,, \end{aligned} \tag{S.39}$$

such that

$$M[\omega_+, \omega_+, g_+]\,(|b|, \epsilon) = \frac{1}{2} \int \mathrm{d}\tau_1 \mathrm{d}\tau_2\, f(\tau_1) f(\tau_1) G(\tau_1 - \tau_2)\,. \tag{S.40}$$

We would like to put $M[\omega_-, \omega_-, g_+]$ in a similar form:

$$\begin{aligned} M[\omega_-, \omega_-, g_+]\,(|b|, \epsilon) &= \frac{1}{2} \int \mathrm{d}\tau_1 \mathrm{d}\tau_2\, f(-\tau_1) f(-\tau_1) G(\tau_1 - \tau_2) \\ &= \frac{1}{2} \int \mathrm{d}\tau_1 \mathrm{d}\tau_2\, f(\tau_2) f(\tau_2) G(\tau_1 - \tau_2)\,, \end{aligned} \tag{S.41}$$

where in the last step we have changed variables $\tau_1 \to -\tau_2$, $\tau_2 \to -\tau_1$. Then define the symmetric quantity:

$$\begin{aligned} B &= M[\omega_+, \omega_+, g_+]\,(|b|, \epsilon) + M[\omega_-, \omega_-, g_+]\,(|b|, \epsilon) \\ &= \frac{1}{2} \int \mathrm{d}\tau_1 \mathrm{d}\tau_2\, \left(f^2(\tau_1) + f^2(\tau_2)\right) G(\tau_1 - \tau_2)\,. \end{aligned} \tag{S.42}$$

By computation, it is found that

$$f(\tau) = \frac{c_0}{\epsilon} + c_1(\tau) + \mathcal{O}(\epsilon)\,, \tag{S.43}$$

and that $G(\tau)$ stars at order ϵ^1. Therefore, expanding A and B yields:

$$\begin{aligned} A &= \int \mathrm{d}\tau_1 \mathrm{d}\tau_2 \left(\frac{c_0^2}{\epsilon^2} + \frac{c_0}{\epsilon}\left(c_1(\tau_1) + c_1(\tau_2)\right) + \mathcal{O}(\epsilon)\right) G(\tau_1 - \tau_2) \\ &= \int \mathrm{d}\tau_1 \mathrm{d}\tau_2 \left(\frac{c_0^2}{\epsilon^2} + 2\frac{c_0}{\epsilon} c_1(\tau_1)\right) G(\tau_1 - \tau_2) + \mathcal{O}(\epsilon)\,, \\ B &= \frac{1}{2} \int \mathrm{d}\tau_1 \mathrm{d}\tau_2 \left(\frac{c_0^2}{\epsilon^2} + 2\frac{c_0}{\epsilon} c_1(\tau_1) + \frac{c_0^2}{\epsilon^2} + 2\frac{c_0}{\epsilon} c_1(\tau_2) + \mathcal{O}(\epsilon)\right) G(\tau_1 - \tau_2) \\ &= \int \mathrm{d}\tau_1 \mathrm{d}\tau_2 \left(\frac{c_0^2}{\epsilon^2} + 2\frac{c_0}{\epsilon} c_1(\tau_1)\right) G(\tau_1 - \tau_2) + \mathcal{O}(\epsilon)\,. \end{aligned} \tag{S.44}$$

Moreover, in position space, we have $A = B + \mathcal{O}(\epsilon)$. In this case, the Fourier transform between position space and momentum space introduces a factor $1/\epsilon$. Thus, the final statement in momentum space is:

$$M[\omega_+, g_+, \omega_-] = -M[\omega_+, \omega_+, g_+] - M[\omega_-, \omega_-, g_+] + \mathcal{O}(\epsilon^0)\,. \tag{S.45}$$

As similar analysis confirms an equivalent relation for the odd-in-v^μ Brandenburg Gate integrals:

$$M[\omega_+, g_+\omega_+, \omega_-] = -M[\omega_+, \omega_+, g_+\omega_+] - M[\omega_-, \omega_-, g_+\omega_+] + \mathcal{O}(\epsilon^0)\,. \tag{S.46}$$

By supplementing the calculation of cuts with these identities, we can determine the series expansion of Brandenburg Gate integrals with all worldline orientations up to $\mathcal{O}(\epsilon^0)$.

VI. SUPPLEMENTARY EQUATIONS

A. Series expansions of boundary integrals

Here we provide all boundary integrals used in our calculation as a series expansion in ϵ. Each integral has been normalized by a factor of $\exp(4\gamma_E\epsilon)\pi^{-4\epsilon}$ for brevity. Additionally, the integrals also depend trivially on γ: for each g, we pull out a factor of $(\gamma^2-1)^{\frac{1}{2}-\epsilon}$, and for each ω a factor of $1/\sqrt{\gamma^2-1}$. In addition, we put $|q| = 1$. We have computed:

PPR Integrals

Even in v^μ

$$M[0, g_+, 0] = -\frac{1}{65536\pi^2} - \frac{(3+26\log(2))\epsilon}{65536\pi^2} + \mathcal{O}(\epsilon^2) \tag{S.47a}$$

$$M[g_+, 0, 0] = -\frac{1}{8192\pi^4} - \frac{(17+6\log(2))\epsilon}{8192\pi^4} + \mathcal{O}(\epsilon^2) \tag{S.47b}$$

$$M[0, g_+\omega_+, \omega_-] = -\frac{1}{4096\pi^4\epsilon^2} - \frac{6+3\log(2)}{2048\pi^4\epsilon} + \mathcal{O}(\epsilon^0) \tag{S.47c}$$

$$M[0, g_+\omega_+, \omega_+] = \frac{1}{4096\pi^4\epsilon^2} + \frac{6+3\log(2)}{2048\pi^4\epsilon} + \mathcal{O}(\epsilon^0) \tag{S.47d}$$

$$M[g_+, \omega_-, \omega_-] = -\frac{3}{16384\pi^4\epsilon^2} - \frac{13+9\log(2)}{8192\pi^4\epsilon} + \mathcal{O}(\epsilon^0) \tag{S.47e}$$

$$M[g_+, \omega_-, \omega_+] = \frac{5}{16384\pi^4\epsilon^2} + \frac{19+15\log(2)}{8192\pi^4\epsilon} + \mathcal{O}(\epsilon^0) \tag{S.47f}$$

$$M[g_+, \omega_+, \omega_-] = \frac{1}{16384\pi^4\epsilon^2} + \frac{-1+3\log(2)}{8192\pi^4\epsilon} + \mathcal{O}(\epsilon^0) \tag{S.47g}$$

$$M[g_+, \omega_+, \omega_+] = \frac{1}{16384\pi^4\epsilon^2} + \frac{7+3\log(2)}{8192\pi^4\epsilon} + \mathcal{O}(\epsilon^0) \tag{S.47h}$$

$$M[\omega_+, g_+, \omega_-] = \frac{1}{8192\pi^4\epsilon^2} + \frac{3+3\log(2)}{4096\pi^4\epsilon} + \mathcal{O}(\epsilon^0) \tag{S.47i}$$

$$M[\omega_+, g_+, \omega_+] = \frac{1}{8192\pi^4\epsilon^2} + \frac{7+3\log(2)}{4096\pi^4\epsilon} + \mathcal{O}(\epsilon^0) \tag{S.47j}$$

$$M[g_+\omega_+, 0, \omega_-] = -\frac{1}{4096\pi^4\epsilon^2} - \frac{6+3\log(2)}{2048\pi^4\epsilon} + \mathcal{O}(\epsilon^0) \tag{S.47k}$$

$$M[g_+\omega_+, 0, \omega_+] = \frac{1}{4096\pi^4\epsilon^2} + \frac{6+3\log(2)}{2048\pi^4\epsilon} + \mathcal{O}(\epsilon^0) \tag{S.47l}$$

$$M[g_+\omega_+, \omega_-, 0] = \frac{1}{8192\pi^2\epsilon} + \mathcal{O}(\epsilon^0) \tag{S.47m}$$

$$M[g_+\omega_+, \omega_+, 0] = -\frac{1}{8192\pi^2\epsilon} + \mathcal{O}(\epsilon^0) \tag{S.47n}$$

Odd in v^μ

$$M[0,g_+,\omega_-] = \frac{i}{768\pi^3} + \frac{55i\epsilon}{2304\pi^3} + \mathcal{O}\big(\epsilon^2\big) \tag{S.48a}$$

$$M[0,g_+,\omega_+] = \frac{i(-1+4\log(2))\epsilon}{768\pi^3} + \mathcal{O}\big(\epsilon^2\big) \tag{S.48b}$$

$$M[0,g_+\omega_+,0] = \frac{i}{1024\pi^3} + \frac{i(4+2\log(2))\epsilon}{256\pi^3} + \mathcal{O}\big(\epsilon^2\big) \tag{S.48c}$$

$$M[g_+,0,\omega_-] = \frac{i}{1024\pi^3} + \frac{i(25+2\log(2))\epsilon}{1536\pi^3} + \mathcal{O}\big(\epsilon^2\big) \tag{S.48d}$$

$$M[g_+,0,\omega_+] = -\frac{i}{3072\pi^3} + \frac{i(-41+30\log(2))\epsilon}{4608\pi^3} + \mathcal{O}\big(\epsilon^2\big) \tag{S.48e}$$

$$M[g_+,\omega_-,0] = \frac{i}{2048\pi^3} + \frac{i(2+3\log(2))\epsilon}{512\pi^3} + \mathcal{O}\big(\epsilon^2\big) \tag{S.48f}$$

$$M[g_+,\omega_+,0] = \frac{i}{2048\pi^3} + \frac{i(4+3\log(2))\epsilon}{512\pi^3} + \mathcal{O}\big(\epsilon^2\big) \tag{S.48g}$$

$$M[g_+\omega_+,0,0] = \frac{i}{1024\pi^3} + \frac{i(7+6\log(2))\epsilon}{512\pi^3} + \frac{i\left(272+9\pi^2+48\log(2)(7+3\log(2))\right)\epsilon^2}{2048\pi^3} + \mathcal{O}\big(\epsilon^3\big) \tag{S.48h}$$

$$M[\omega_+,g_+\omega_+,\omega_-] = -\frac{i}{2048\pi^3\epsilon^2} - \frac{i(1+2\log(2))}{1024\pi^3\epsilon} + \mathcal{O}\big(\epsilon^0\big) \tag{S.48i}$$

$$M[\omega_+,g_+\omega_+,\omega_+] = \frac{i}{2048\pi^3\epsilon^2} + \frac{i(1+2\log(2))}{1024\pi^3\epsilon} + \mathcal{O}\big(\epsilon^0\big) \tag{S.48j}$$

$$M[g_+\omega_+,\omega_-,\omega_-] = \frac{i}{4096\pi^3\epsilon^2} + \frac{i(1+2\log(2))}{2048\pi^3\epsilon} + \mathcal{O}\big(\epsilon^0\big) \tag{S.48k}$$

$$M[g_+\omega_+,\omega_-,\omega_+] = -\frac{3i}{4096\pi^3\epsilon^2} - \frac{3i(1+2\log(2))}{2048\pi^3\epsilon} + \mathcal{O}\big(\epsilon^0\big) \tag{S.48l}$$

$$M[g_+\omega_+,\omega_+,\omega_-] = -\frac{3i}{4096\pi^3\epsilon^2} - \frac{3i(1+2\log(2))}{2048\pi^3\epsilon} + \mathcal{O}\big(\epsilon^0\big) \tag{S.48m}$$

$$M[g_+\omega_+,\omega_+,\omega_+] = \frac{i}{4096\pi^3\epsilon^2} + \frac{i(1+2\log(2))}{2048\pi^3\epsilon} + \mathcal{O}\big(\epsilon^0\big) \tag{S.48n}$$

PRR Integrals

Even in v^μ

$$M[g_-g_+,0] = \frac{1}{30720\pi^4\epsilon} + \frac{58+15\log(2)}{115200\pi^4} + \mathcal{O}\big(\epsilon^1\big) \tag{S.49a}$$

$$M[g_+g_+,0] = -\frac{1}{30720\pi^4\epsilon} - \frac{58+15\log(2)}{115200\pi^4} + \mathcal{O}\big(\epsilon^1\big) \tag{S.49b}$$

$$M[g_-g_+\omega_+,\omega_+] = -\frac{1}{4096\pi^4\epsilon} - \frac{7+2\log(2)}{2048\pi^4} + \mathcal{O}\big(\epsilon^1\big) \tag{S.49c}$$

$$M[g_+g_+\omega_+,\omega_-] = \frac{3}{4096\pi^4\epsilon} + \frac{17+6\log(2)}{2048\pi^4} + \mathcal{O}\big(\epsilon^1\big) \tag{S.49d}$$

$$M[g_+g_+\omega_+,\omega_+] = -\frac{1}{4096\pi^4\epsilon} - \frac{3+2\log(2)}{2048\pi^4} + \mathcal{O}\big(\epsilon^1\big) \tag{S.49e}$$

Odd in v^μ

$$M[g_-g_+,\omega_+] = \frac{i}{8192\pi^3\epsilon} + \frac{i(7+10\log(2))}{8192\pi^3} + \mathcal{O}\big(\epsilon^1\big) \tag{S.50a}$$

$$M[g_+g_+,\omega_-] = \frac{i}{8192\pi^3\epsilon} + \frac{i(7+10\log(2))}{8192\pi^3} + \frac{i\left(342+5\pi^2+60\log(2)(7+5\log(2))\right)\epsilon}{49152\pi^3} + \mathcal{O}\big(\epsilon^2\big) \tag{S.50b}$$

$$M[g_+g_+,\omega_+] = -\frac{i}{8192\pi^3\epsilon} - \frac{i(7+10\log(2))}{8192\pi^3} - \frac{i\left(342+29\pi^2+60\log(2)(7+5\log(2))\right)\epsilon}{49152\pi^3} + \mathcal{O}\big(\epsilon^2\big) \tag{S.50c}$$

$$M[g_-g_+\omega_+,0] = 0 \tag{S.50d}$$

$$M[g_+g_+\omega_+,0] = -\frac{i\epsilon}{384\pi^3} + \frac{i(-34+9\log(2))\epsilon^2}{576\pi^3} + \mathcal{O}\big(\epsilon^3\big) \tag{S.50e}$$

RRR Integrals

Even in v^μ

$$M[g_-g_+g_+] = -\frac{1}{12288\pi^4} - \frac{(41+6\log(2))\epsilon}{36864\pi^4} + \mathcal{O}\big(\epsilon^2\big) \tag{S.51a}$$

$$M[g_+g_+g_+] = \frac{1}{4096\pi^4} + \frac{(41+6\log(2))\epsilon}{12288\pi^4} + \frac{\big(587-6\pi^2+3\log(2)(41+3\log(2))\big)\epsilon^2}{18432\pi^4} + \mathcal{O}\big(\epsilon^3\big) \tag{S.51b}$$

$$M[\text{tail}_+\text{tail}_+] = -\frac{3}{4096\pi^4\epsilon} - \frac{3(8+\log(2))}{2048\pi^4} - \frac{3(90+\log(2)(16+\log(2)))\epsilon}{2048\pi^4} + \mathcal{O}\big(\epsilon^2\big) \tag{S.51c}$$

$$M[\text{tail}_+\text{tail}_-] = -\frac{1}{4096\pi^4\epsilon} - \frac{8+\log(2)}{2048\pi^4} + \mathcal{O}\big(\epsilon^1\big) \tag{S.51d}$$

Odd in v^μ

$$M[g_-g_+g_+\omega_+] = \frac{i}{4096\pi^3} + \frac{i(1+4\log(2))\epsilon}{1024\pi^3} + \mathcal{O}\big(\epsilon^2\big) \tag{S.52a}$$

$$M[g_+g_+g_+\omega_+] = -\frac{i}{4096\pi^3} - \frac{i(1+4\log(2))\epsilon}{1024\pi^3} + \mathcal{O}\big(\epsilon^2\big) \tag{S.52b}$$

$$M[\text{tail}_+\text{tail}_+\omega_+] = \frac{i}{1024\pi^3\epsilon} + \frac{i(1+2\log(2))}{128\pi^3} + \mathcal{O}\big(\epsilon^1\big) \tag{S.52c}$$

$$M[\text{tail}_+\text{tail}_-\omega_+] = 0 \tag{S.52d}$$

The PPP boundary integrals can be decomposed into simple products of Γ-functions that can be obtained from the loop-by-loop methods given above.

B. Additional G_i functions

We list here all 20 new transcendental functions necessary to derive the canonical form of the differential equations:

$$G_1'(x) = -\frac{96x\left(x^4+1\right)\varpi_0(x)^2}{(x-1)^2(x+1)^2\left(x^2+1\right)^2\alpha_1(x)}\,, \tag{S.53a}$$

$$\begin{aligned}G_2'(x) = &-\frac{16\left(7x^{12}+314x^{10}+329x^8+1340x^6+329x^4+314x^2+7\right)\varpi_0(x)^2}{3(x-1)^3x(x+1)^3\left(x^2+1\right)^3}\\ &+\frac{16\left(7x^8+136x^6+42x^4+136x^2+7\right)\varpi_0(x)^2\alpha_1'(x)}{3(x-1)^2(x+1)^2\left(x^2+1\right)^2\alpha_1(x)} - \frac{2\left(5x^8+28x^6+262x^4+28x^2+5\right)\varpi_0(x)^2\alpha_1'(x)^2}{3(x-1)x(x+1)\left(x^2+1\right)\alpha_1(x)^2}\\ &-\frac{xG_1(x)^2\alpha_1(x)^2}{(x-1)(x+1)\left(x^2+1\right)\varpi_0(x)^2}\,,\end{aligned} \tag{S.53b}$$

$$G_3'(x) = -\frac{xG_1(x)\alpha_1(x)^2}{(x-1)(x+1)\left(x^2+1\right)\varpi_0(x)^2}\,, \tag{S.53c}$$

$$\begin{aligned}G_4'(x) = &-\frac{16\left(7x^8+136x^6+42x^4+136x^2+7\right)\varpi_0(x)^2}{3(x-1)^2x(x+1)^2\left(x^2+1\right)^2\alpha_1(x)} + \frac{4\left(5x^8+28x^6+262x^4+28x^2+5\right)\varpi_0(x)^2\alpha_1'(x)}{3(x-1)x^2(x+1)\left(x^2+1\right)\alpha_1(x)^2}\\ &+\frac{G_2(x)}{x\alpha_1(x)}\,,\end{aligned} \tag{S.53d}$$

$$G_5'(x) = \varpi_{0,\mathrm{K3}}(x)\varpi_0''(x)\,, \tag{S.53e}$$

$$G_6'(x) = \varpi_{0,\mathrm{K3}}(x)\varpi_0'(x)\,, \tag{S.53f}$$

$$G_7'(x) = \frac{\varpi_{0,\mathrm{K3}}(x)G_3(x)\varpi_0'(x)}{\alpha_1(x)}\,, \tag{S.53g}$$

$$G_8'(x) = \frac{\varpi_{0,\mathrm{K3}}(x) G_3(x) \varpi_0(x) \alpha_1'(x)}{\alpha_1(x)^2}\,, \tag{S.53h}$$

$$G_9'(x) = \frac{\varpi_{0,\mathrm{K3}}(x) \varpi_0'(x)}{\alpha_1(x)} - \frac{\varpi_{0,\mathrm{K3}}(x) \varpi_0(x) \alpha_1'(x)}{2\alpha_1(x)^2}\,, \tag{S.53i}$$

$$G_{10}'(x) = -\frac{\left(3x^4-2x^2+3\right)\varpi_{0,\mathrm{K3}}(x)\varpi_0(x)}{32x^2} + \frac{4x^3\left(3x^2-5\right)G_5(x)}{32x^2} + \frac{(27x^4-10x^2+3)G_6(x)}{32x^2}\,, \tag{S.53j}$$

$$G_{11}'(x) = -\frac{3(x-1)(x+1)\left(x^2+1\right)\varpi_{0,\mathrm{K3}}(x)\varpi_0(x)}{8x^2} + \frac{12x^5 G_5(x)}{8x^2} + \frac{(27x^4-4x^2-3)G_6(x)}{8x^2}\,, \tag{S.53k}$$

$$\begin{aligned}G_{12}'(x) = &-\frac{(x^{12}+2x^{10}-73x^8+236x^6-73x^4+2x^2+1)\varpi_{0,\mathrm{K3}}(x)\varpi_0(x)}{16(x-1)^2x^2(x+1)^2\left(x^2+1\right)^2} - \frac{\left(x^2-3\right)\left(3x^2-1\right)\varpi_{0,\mathrm{K3}}(x)G_1(x)\alpha_1(x)}{32(x-1)(x+1)\left(x^2+1\right)\varpi_0(x)}\\
&+\left[\frac{3(x-1)(x+1)\left(x^2+1\right)\varpi_{0,\mathrm{K3}}(x)\varpi_0(x)}{16x^2\alpha_1(x)} - \frac{\left(x^2-3\right)\left(3x^2-1\right)G_5(x)}{32x\alpha_1(x)} - \frac{(9x^4-10x^2-3)G_6(x)}{32x^2\alpha_1(x)}\right]G_3(x)\\
&+\frac{\left(x^2-3\right)\left(3x^2-1\right)\left(x^4-16x^2+1\right)G_5(x)}{48(x-1)x(x+1)\left(x^2+1\right)} + \frac{(9x^{12}-143x^{10}-85x^8+624x^6-551x^4+47x^2+3)G_6(x)}{24(x-1)^2x^2(x+1)^2\left(x^2+1\right)^2}\\
&-\frac{(9x^4-10x^2-3)G_7(x)}{16x^2} + \frac{(9x^4-10x^2-3)G_8(x)}{32x^2} - \frac{2\left(x^4-16x^2+1\right)G_{10}(x)}{3(x-1)x(x+1)\left(x^2+1\right)}\end{aligned} \tag{S.53l}$$

$$\begin{aligned}G_{13}'(x) = &-\frac{15\left(x^2+1\right)\varpi_0(x)}{(x-1)^2(x+1)^2} - \frac{1}{\varpi_{0,\mathrm{K3}}(x)}\left[\frac{13\left(x^2-3\right)\left(3x^2-1\right)G_5(x)}{6(x-1)x(x+1)} + \frac{13\left(9x^4-10x^2-3\right)G_6(x)}{6(x-1)x^2(x+1)}\right.\\
&\left.-\frac{208G_{10}(x)}{3(x-1)x(x+1)}\right]\,,\end{aligned} \tag{S.53m}$$

$$G_{14}'(x) = -\frac{1}{\varpi_{0,\mathrm{K3}}(x)}\left[-\frac{3\left(x^2+1\right)G_5(x)}{4x} - \frac{(9x^4-4x^2+3)G_6(x)}{4(x-1)x^2(x+1)} + \frac{2G_{11}(x)}{(x-1)x(x+1)}\right]\,, \tag{S.53n}$$

$$\begin{aligned}G_{15}'(x) = &-\frac{(x^8-12x^6-2x^4-12x^2+1)\varpi_{0,\mathrm{K3}}(x)\varpi_0(x)}{4(x-1)x^2(x+1)\left(x^2+1\right)} - \frac{3\varpi_{0,\mathrm{K3}}(x)G_1(x)\alpha_1(x)}{8\varpi_0(x)}\\
&+\left[\frac{(3x^4-2x^2+3)\varpi_{0,\mathrm{K3}}(x)\varpi_0(x)}{4x^2\alpha_1(x)} - \frac{3(x-1)(x+1)\left(x^2+1\right)G_5(x)}{8x\alpha_1(x)} - \frac{(9x^4-4x^2+3)G_6(x)}{8x^2\alpha_1(x)}\right.\\
&\left.+\frac{G_{11}(x)}{x\alpha_1(x)}\right]G_3(x) + \frac{(x^4-16x^2+1)G_5(x)}{4x} + \frac{(9x^8-89x^6+32x^4-11x^2+3)G_6(x)}{6(x-1)x^2(x+1)\left(x^2+1\right)} - \frac{3\left(3x^4+1\right)G_7(x)}{4x^2}\\
&+\frac{3\left(3x^4+1\right)G_8(x)}{8x^2} - \frac{2\left(x^4-16x^2+1\right)G_{11}(x)}{3(x-1)x(x+1)\left(x^2+1\right)}\,,\end{aligned} \tag{S.53o}$$

$$\begin{aligned}G_{16}'(x) = &-\frac{3(x-1)(x+1)\left(x^2+1\right)\varpi_{0,\mathrm{K3}}(x)\varpi_0(x)}{16x^2\alpha_1(x)} + \frac{\left(x^2-3\right)\left(3x^2-1\right)G_5(x)}{32x\alpha_1(x)} + \frac{(9x^4-10x^2-3)G_6(x)}{32x^2\alpha_1(x)}\\
&+\frac{(9x^4-10x^2-3)G_9(x)}{16x^2} - \frac{G_{10}(x)}{x\alpha_1(x)}\,,\end{aligned} \tag{S.53p}$$

$$\begin{aligned}G_{17}'(x) = &-\frac{\left(3x^4-2x^2+3\right)\varpi_{0,\mathrm{K3}}(x)\varpi_0(x)}{4x^2\alpha_1(x)} - \frac{3(x-1)(x+1)\left(x^2+1\right)G_5(x)}{8x\alpha_1(x)} + \frac{(9x^4-4x^2+3)G_6(x)}{8x^2\alpha_1(x)}\\
&+\frac{3\left(3x^4+1\right)G_9(x)}{4x^2} - \frac{G_{11}(x)}{x\alpha_1(x)}\,,\end{aligned} \tag{S.53q}$$

$$G_{18}'(x) = \frac{\varpi_0(x)}{(x-1)^2}\,, \tag{S.53r}$$

$$G_{19}'(x) = \frac{\varpi_0(x)}{x}\,, \tag{S.53s}$$

$$G_{20}'(x) = \frac{\varpi_0(x)}{(x+1)^2}\,, \tag{S.53t}$$

where $\varpi_{0,\mathrm{K3}} = (\frac{2}{\pi})^2 K^2(1-x^2)$ and $f'(x) = \frac{\mathrm{d}}{\mathrm{d}x} f(x)$.

---

[1] Driesse, M., Uhre Jakobsen, G., Klemm, A., Mogull, G., Nega, C., Plefka, J., Sauer, B. & Usovitsch, J., High-precision black hole scattering with Calabi-Yau manifolds. Zenodo., (2024). https://doi.org/10.5281/zenodo.14604438.

[2] Wasow, W. Asymptotic simplification of self-adjoint differential equations with a parameter. *Journal of Differential Equations* **2**, 378–390 (1966). URL https://www.sciencedirect.com/science/article/pii/0022039666900489.

[3] Lee, R. N. Libra: A package for transformation of differential systems for multiloop integrals. *Comput. Phys. Commun.* **267**, 108058 (2021). 2012.00279.

[4] Henn, J., Mistlberger, B., Smirnov, V. A. & Wasser, P. Constructing d-log integrands and computing master integrals for three-loop four-particle scattering. *JHEP* **04**, 167 (2020). 2002.09492.

[5] Dlapa, C., Kälin, G., Liu, Z. & Porto, R. A. Bootstrapping the relativistic two-body problem. *JHEP* **08**, 109 (2023). 2304.01275.

[6] Edison, A. & Levi, M. Higher-order tails and RG flows due to scattering of gravitational radiation from binary inspirals. *JHEP* **08**, 161 (2024). 2310.20066.

[7] M. Driesse, G. U. Jakobsen, G. Mogull, J. Plefka, B. Sauer and J. Usovitsch, Phys. Rev. Lett. **132** (2024) no.24, 241402 doi:10.1103/PhysRevLett.132.241402 [arXiv:2403.07781 [hep-th]].

[8] C. Duhr and F. Dulat, JHEP **08**, 135 (2019) doi:10.1007/JHEP08(2019)135 [arXiv:1904.07279 [hep-th]].
